# Supplementary material for: Experimental Infection of Newly Hatched Domestic Ducklings via Japanese Encephalitis Virus-Infected Mosquitoes
Source: Pathogens. 2020 May 12;9(5):371. doi: 10.3390/pathogens9050371 (PMC7281460; doi:10.3390/pathogens9050371)
Supplement: Supplementary file 1 [file pathogens-09-00371-s001.zip › supplement materials/Figure S2.docx]

**Figure S2 Nucleotide and amino acid sequences of JEV recovered from the affected duckling brains**

**(a) Nucleotide sequence**

agaagtttatctgtgtgaacttcttggcttagtatcgttgagaagaatcgagagattagtgcagtttaaacagttttttagaacggaagaacaaccatgactaaaaaaccaggagggcccggaaaaaaccgggccatcaatatgctgaaacgcggattaccccgcgtattcccactagtgggagtgaagagggtagttatgagcttgttggacggcagagggccagtacgatttgtgctggctcttatcacgttcttcaagtttacagcattagccccgaccaaggcgcttttgggccgctggagagcagtggaaaaaagtgtggcaatgaaacatcttaccagtttcaaacgagaacttggaacactcattgacgccgtgaacaagcggggcaaaaaacaaaacaaaagaggagggaatgaaagcttgattatgtggcttgccagcttggcaatcgtaacagcctgtgccggagccatgaagctatcaaactttcaaggaaagcttctgatgaccatcaacaacacggacattgcggatgtcatcgtgatccccacctcaaaaggtgaaaacagatgctgggtacgagcaatcgacgttggttacatgtgtgaagacaccatcacgtatgaatgtccgaagcttgccgtgggcaacgatccggaagacgtggactgctggtgcgacaatcaggaagtctacgtgcagtatggtcgctgcacacggaccaggcattccaaacgaagcagaagatccgtttcggtccacacgcatggggaaagctcactagtgaacaaaaaagaggcttggctggattcaacgaaggccacgcgatacctcatgaaaacggagaactggatcataaggaaccctggttatgctttcctggcggtggcacttggatggatgcttggcagcaacaatggccaacgtgtggtgttcactattctcttgctattggtcgctccggcttacagttttaactgtctgggaatggggaatcgggatttcatagaaggagccagtggagccacttgggtggatctggtgttggaaggagatagctgtttgacaattatggcaaacgacaaaccaacactagatgtccgcatgatcaacattgaagctagccaacttgctgaagtcaggagttactgctatcacgcttcagtcactgacatttcaacggtggctcgatgccccacgactggagaagcccacaatgaaaaacgtgctgacagcagctacgtgtgcaaacagggctttactgaccgcggatggggaaatggatgtggacttttcgggaaaggaagcattgacacatgcgcaaaattttcttgtaccagtaaggccattggaagaatgatccaaccagagaacatcaagtacgcggttggcatattcgtgcacggaaccactacctcggaaaaccacgggaattactcagcgcaagtaggagcgtctcaagcagcaaagtttactgtaactccaaatgctccttcaataaccctcaagcttggtgattatggagaagtcacactggattgtgaaccaaggagtggactgaacactgaagcgttctatgtcatgaccgtgggttcgaagtcattcttagtccatagggaatggttccatgacctttctcttccctggacgtccccctcaagcacggcatggagaaacagagaactcctcatggaatttgaagaggcacatgccacaaaacaatctgtcgtagctcttgggtcacaggagggaggcctccatcaagcgttggcaggagccatcgtggtggagtactcgagctcagtgaaattgacatcaggtcacctgaaatgcaggctaaaaatggacaaactggctctgaagggcacgacttatggcatgtgtacagaaaaattctcgttcgcgaaaaatccagcggacacgggccatggaacagttgtcattgagctcacatattctggaagtgatggtccctgtaaaattccgattgtctcagtcgctagtttaaacgacatgacccctgtggggaggctggtaacagtaaaccccttcgtcgcaacatctagctccaactcaaaggtgctggttgagatggaacctcccttcggagactcttatatcgtggttggaagaggggacaagcagattaaccatcactggcacaaagctggaagcacgctgggcaaagctttctcaacaactttgaaaggggctcagagactagcagcgctaggtgacacagcctgggacttcggttccattggaggggtattcaactccatagggaaagctgttcaccaagtatttggcggtgcattcagaacgctctttgggggaatgtcttggatcacacaaggactaatgggggccttacttctttggatgggtgtcaacgcacgagaccggtcaatcgccctggcttttctggccacgggaggtgtgctcgtgtttttggcgaccaatgtgcatgccgacactggctgtgccattgacatcacaagaaaagagatgaggtgtggaagtggcatcttcgtgcacaacgacgtagaggcttgggtagataggtacaaatatttgccagagacgcccagatccttagcgaagatcgtccacaaagcacatcaagaaggagtgtgcggggtcagatccgtcactagactggaacaccagatgtgggagtctgtgcgggacgaactgaatgtcttgctcaaagagaacgcggtggatctcagtgtggtggtgaacaagcccgtggggagatatcgctcagcccccaaacgcctatccatgactcaagaaaagtttgagatgggctggaaagcatggggaaaaagcattctcttcgcccccgaattggccaactccacgttcgtcgtggatggacccgagacaaaggaatgccctgatgagcgcagagcctggaacagcatgcaaatcgaagatttcggcttcggcatcacatcaacccgagtgtggctgaaaattagagaggagaacactgatgggtgtgatggagcaatcatagggacagctgttaaagggcatgtggcagttcatagtgacttgtcatactggatcgagagccgtctcaatgacacctggaaacttgagagggctgtcttcggagaggtgaaatcttgcacttggcccgagacacacactctttggggtgacggtgttgaggagagcgagcttatcatcccacacaccatagctggaccgagaagcaagcacaaccggagagaagggtataaaacacaaaaccagggaccctgggatgagaacggcatcgttcttgactttgactattgtccaggaacaaaagtcaccatcacagaggactgtggcaagaggggtccctcaatcagaaccactactgacagtggaaagctgatcaccgattggtgctgccgcagctgttctctaccgcctttgcggttccggacagaaaatggttgctggtatgggatggaaatcagacctgttaggcatgacgaaacaacactcgttaggtcacaggttgacgctttcaacggcgaaatgattgacccttttcagttgggccttctggtgatgtttctggccacccaggaggtccttcgcaagaggtggacggccagattgacgattcctgcggttttgggggctctacttgtgctgatgcttgggggcatcacttacactgacctggcaagatatgtggtgctagttgctgcggctttcgcggaggccaacagtggaggagatgttctgcacctcgctctgatagccgtcttcaagatccaaccagcttttctggtcatgaacatgcttagcgcgagatggacgaaccaagagaacgtggttctggtcctgggggcggcttttttccaactagcttcagtggatttacagatcggagtccacggaatcctgaatgccgcagccatagcatggatgatcgttcgagcgatcacatttcccacaacttctaccgttgccatgccaatcttagcgctcctaactccgggaatgagggctttgtatctggacacttacagaatcattcttcttgtcataggaatttgttccctgctgcaagagaggagaaagaccatggcgaagaagaaaggagccgtgctcttgggcttagcgctcacatccaccggatggttttcgcccaccactatcgcagccggactaatggtctgcaacccaaacaagaagagagggtggccagccaccgagttcctttcagcggttgggttgatgtttgccattgtgggaggtctagccgagttggacatcgaatctatgtcaatacccttcatgctggcagggctcatggcagtgtcctacgtggtatcaggaaaggcaaccgacatgtggctggaccgggctgccgatatcagctgggagatggaggctgcaatcacaggaagtagccggaggctagatgttaagttggatgacgacggcgactttcacttgattgatgatcccggtgttccatggaaagtctggcttttgcgcatgtcttgtatcggtttagccgctctgacaccctgggctatcgttcccgccgcttttggctattggctcactctgaaaacaacaaaaagagggggcgtgttctgggacacgccatctccaaagccttgcttaaagggggacaccaccacaggagtttaccgaattatggccagggggattctaggcacttaccaggccggagttggagtcatgtatgagaatgttttccacacattgtggcacacaactagaggagcagccatcatgagtggagaaggaaagctaacgccatactggggtagtgtgaaggaagaccgcataagctatggaggcccgtggaggtttgaccggaagtggaatggaacagatgatgtgcaagtgattgtggtggaaccagggaaacctgcagtaaacatccagacaaaaccgggagtgtttcgcacccctttcggggaggttggagcagtcagcttggactacccacggggaacatccggctcacccatcctagattccaatggagacatcataggcttgtatggcaatggggttgaactcggcgatggatcgtatgtcagcgccattgtgcagggcgaccgtcaagaggaaccagttccagatgcctacactccaagcatgctgaaaaagagacagatgactgtgctggacctgcacccaggttcgggaaaaaccaggaagatcctaccccaaataattaaggacgccatccagcagcgcttgagaacagccgtgctggcacccacacgagtggtagcagcagaaatggcggaagctttgagaggactcccagtacgatatcaaacctcggcagtgcagagggagcatcagggaaatgaaatagtagacgtaatgtgccatgccactctgacccatagactaatgtcaccaaacagggtgcccaactacaatttattcgtgatggatgaggctcacttcactgacccagctagcatcgccgctcggggttatattgcaaccaaggtggaactgggggaggcagcagccatttttatgacggcgaccccgcccgggaccactgatccctttcccgactcaaatgccccaattcatgacctgcaggatgagatcccagacagggcatggagcagtggatacgaatggatcacggactatgcgggaaaaactgtgtggttcgtggcaagtgtgaaaatggggaatgagatcgcaatgtgcctccaaagagcggggaaaaaggtcatccagctcaatcgtaagtcatatgacacagaatacccaaaatgcaaaaatggagattgggactttgttatcaccactgacatctctgagatgggggccaattttggtgcgagcagggtcattgactgcagaaagagtgtgaaacccaccatcctagaggagggagaaggtagagtcattcttggaaacccatcccccataaccagtgcaagtgcagcccaacggagaggtagagtgggcaggaatcccaaccaagttggagatgaataccattacgggggggccaccagtgaagatgacagcaacctagcccactggacagaggcaaaaattatgctagacaacatacatatgcctaatggattagtggctcagctgtacgggccagagagggaaaaggctttcacaatggatggagagtaccgactcagaggtgaggagaagaagaacttcttggagctgcttagaacggctgacctcccagtatggctagcctacaaggtggcgtccaatggcattcagtacactgacagaaaatggtgctttgatggaccacgcacaaatgccatactagaagataacactgaggtggagatagtcacccgaatgggtgagagaaagatcctcaagccgagatggctcgatgcgagggtttatgcagaccaccaggccctcaagtggtttaaggattttgcagcgggcaagagatcagccgtcagtttcatagaggtgctcggtcgcatgcctgagcatttcatgggaaagacacgggaagccttagacacaatgtatctggtggcaacagctgagaaaggtggaaaggcacaccgcatggctcttgaagaactgcccgacgcattggagaccatcacactcatcgttgccatcactgtgatgacaggaggattcttcctgctcatgatgcagagaaagggtataggaaaaatgggtctaggggctctagtgctcacgctggccacctttttcctatgggcggcagaggttcctggaaccaaaatagcgggcaccctactggtcgccctgttgctaatggtggtcctcatcccggaaccagaaaaacagaggtcacagacagacaaccagttggcagtgtttcttatctgcgtcctgaccgtggtcggagtggtggcagcaaatgagtacggaatgctggaaaaaaccaaagcagaccttaagagcatgtttggcggaaggacgcaagcaccaggactgaccggattgcctagcatggcactggacttgcgcccagccacagcttgggcgctgtatggggggagcacagttgtgttaacccctctcctgaagcatctaatcacctcagaatatgtcaccacatcgttagcttcaatcagttcacaagcgggttcgctgtttgttttgccgcgaggcgtgcctttcactgacttggatctaaccgttggccttgtctttcttggctgttggggccaaatcaccctcaccacgttcctaacagctatggtgctagtgacactccactatggatacatgctccctgggtggcaagcagaggcactcagagctgctcagagaagaacagcggctggcataatgaagaatgccgtcgtggacggaatggtcgccaccgatgtgcccgaactggaaagaactactcccttgatgcaaaagaaagtcggccaagtgctcctcataggggtcagcgtggcggcgtttctcgtcaaccccaatgtcaccaccgtgagagaggcaggtgtgttggtgacggcagccacactcaccttgtgggacaatggggccagtgccgtctggaattccaccaccgctacggggctttgccatgtcatgcgaggcagctacctagctggcggctccattgcctggactctcatcaagaacgctgacaagccctccttaaaaagggggaggcctggaggcaggacgctaggggagcagtggaaggaaaagttgaatgctatgagcagggatgagttcttcaaatacagaagagaggccataattgaggtggaccgcactgaagcacgcagggctaggcgcgagaacaacatagtgggaggacacccagtctcgcgagggtcagcaaagctccgctggctcgtggaaaaaggatttgtctcgccaataggaaaagtcatagatctgggatgcgggcgcggaggctggagctactacgcagcaactctgaaaaaagttcaggaagtcaaagggtacacgaaaggtggggcgggacacgaagaaccgatgctcatgcagagttacggttggaacctggtctcgttaaagagtggggtggacgtattctacaaaccctcggagcctagtgacaccctgttctgtgacataggagaatcttccccaagtccagaggtggaggaacaacgcacgctgcgcgtcctagaaatgacatctgactggttacatcggggacctagagagttctgcataaaagtgctctgcccttacatgcctaaagttatagaaaagatggaagtcctgcaacgtcgtttcggtggcgggttggtgcgccttcccctgtctcgaaactccaaccatgagatgtactgggtgagtggagctgctggcaatgtggtgcatgcggtcaacatgaccagccaagtgctgctagggcgaatggatcgcacagtgtggagagggccaaagtatgaagaggatgtcaacctgggcagcgggacgagagctgtggggaagggagaggtccatagcgaccagaaaaaaattaggaagagaatccagaaacttagagaggaattcgctacaacctggcacaaagaccccgagcatccataccgaacttggacctatcatggaagctacgaagtgaaggccactggctcagcaagctctctcgtcaatggggtggtaaagctcatgagcaaaccctgggacgccatcgccaatgtcaccacaatggccatgaccgacaccaccccctttggccaacagagggtcttcaaagagaaggttgacacgaaggctccagagccaccagcaggagtcaaggaagtgctcaacgagaccaccaactggctgtgggcccacttgtcacgggagaaacgaccccgcttgtgcactaaggaagaattcataaagaaagtcaacagcaacgcagctctcggagcagtgttcgctgaacaaaaccaatggagcacggcgcgggaagccgtgggcgaccctctgttctgggagatggtcaatgaagaaagggaaaaccatttgcgaggggagtgccacacgtgcgtttacaacatgatgggaaaaagagagaaaaaacctggagagttcggaaaggctaaagggagtagggctatttggtttatgtggctcggagctcggtacctagagttcgaagccctaggatttctaaatgaagaccattggctgagccgagagaattcaggaggtggggtggaaggttcaggcgtccaaaagctgggatacattctccgtgacatagcagggaagcaaggaggtaaaatgtatgccgatgacaccgccgggtgggacaccagaatcactagaaccgacttggaaaatgaagccaaagtgctggagcttttggatggtgaacatcgcatgctcgcccgagccataattgaactaacgtacaggcacaaagtggtcaaggttatgaggcctgcagcaggaggaaagacagtgatggacgtgatatcacgagaagaccaaagggggagtgggcaggtggtgacctacgctctcaacacattcacgaacattgctgtccagcttgtccgcttgatggaggctgagggggtcattggaccacaacacttggaacagctgcccaggaaaaacaaaatagctgttaggacctggctctttgagaatggagaggagagagtgactaggatggcgatcagtggagacgactgcgttgtcaagccgctggatgacagattcgccacggctctccatttcctcaacgcaatgtcgaaggtcagaaaagatatccaagaatggaagccttcgcatggttggcacgactggcagcaggtccccttttgctccaatcattttcaggagatcgtgatgaaagatggaaggagcatagtcgtcccgtgcagagggcaggatgagctgattggcagggcgcgcatctccccaggagctggatggaatgtgaaggacacagcttgcctggccaaagcgtatgcacagatgtggctgcttctatacttccatcggagggacctacgccttatggcaaatgcaatctgctcagcagttccagtggactgggtgcccacaggcagaacatcctggtcaatacactcaaaaggagagtggatgaccactgaagacatgctgcaagtctggaacagggtatggattgaagaaaatgaatggatgatggacaagaccccaatcacaagctggacagacgttccgtacgtgggaaagcgtgaggacatctggtgtggcagtctcatcggaacgcgatccagggcaacatgggctgagaacatctacgcggcaataaaccaagtgagggccatcattggaaaagaaaattatgttgattacatgacttccctcagaagatatgaggatgtattgatccaggaggatagggtcatttagacatgataaagtcatgtgtgtaatgtgagacaagaaaatgtgcatgtggagtcaggccagcaaaagctgccaccggatactgagtagacggtgctgcctgcgtctcagtcccaggaggactgggttaacaaatctgacaacggaaggtgggaaagccctcagaaccgtctcggaagcaggtccctgctcaccggaagttgaaagaccaacgtcaggccacaattttgtgccactccgctggggagtgcggcctgcgcagccccaggaggactgggttaacaaagccgttgaggcccccacggcccaagcctcgtctaagatgcaatagactaggtgtaaggactagaggttagaggagaccccgtggaaacaacattatgcggcccaagccccctcgaagctgtagaggaggtggaaggactagaggttagaggagaccccgcatttgcatcaaaacagcatattgacacctgggaatagactgggagatcttctgctctatctcaacatcagctactaggcacagagcgccgaagtatgtagctggtggtgaggaagaacacaggatct

**(b) Amino acid sequence**

MTKKPGGPGKNRAINMLKRGLPRVFPLVGVKRVVMSLLDGRGPVRFVLALITFFKFTALAPTKALLGRWRAVEKSVAMKHLTSFKRELGTLIDAVNKRGKKQNKRGGNESLIMWLASLAIVTACAGAMKLSNFQGKLLMTINNTDIADVIVIPTSKGENRCWVRAIDVGYMCEDTITYECPKLAVGNDPEDVDCWCDNQEVYVQYGRCTRTRHSKRSRRSVSVHTHGESSLVNKKEAWLDSTKATRYLMKTENWIIRNPGYAFLAVALGWMLGSNNGQRVVFTILLLLVAPAYSFNCLGMGNRDFIEGASGATWVDLVLEGDSCLTIMANDKPTLDVRMINIEASQLAEVRSYCYHASVTDISTVARCPTTGEAHNEKRADSSYVCKQGFTDRGWGNGCGLFGKGSIDTCAKFSCTSKAIGRMIQPENIKYAVGIFVHGTTTSENHGNYSAQVGASQAAKFTVTPNAPSITLKLGDYGEVTLDCEPRSGLNTEAFYVMTVGSKSFLVHREWFHDLSLPWTSPSSTAWRNRELLMEFEEAHATKQSVVALGSQEGGLHQALAGAIVVEYSSSVKLTSGHLKCRLKMDKLALKGTTYGMCTEKFSFAKNPADTGHGTVVIELTYSGSDGPCKIPIVSVASLNDMTPVGRLVTVNPFVATSSSNSKVLVEMEPPFGDSYIVVGRGDKQINHHWHKAGSTLGKAFSTTLKGAQRLAALGDTAWDFGSIGGVFNSIGKAVHQVFGGAFRTLFGGMSWITQGLMGALLLWMGVNARDRSIALAFLATGGVLVFLATNVHADTGCAIDITRKEMRCGSGIFVHNDVEAWVDRYKYLPETPRSLAKIVHKAHQEGVCGVRSVTRLEHQMWESVRDELNVLLKENAVDLSVVVNKPVGRYRSAPKRLSMTQEKFEMGWKAWGKSILFAPELANSTFVVDGPETKECPDERRAWNSMQIEDFGFGITSTRVWLKIREENTDGCDGAIIGTAVKGHVAVHSDLSYWIESRLNDTWKLERAVFGEVKSCTWPETHTLWGDGVEESELIIPHTIAGPRSKHNRREGYKTQNQGPWDENGIVLDFDYCPGTKVTITEDCGKRGPSIRTTTDSGKLITDWCCRSCSLPPLRFRTENGCWYGMEIRPVRHDETTLVRSQVDAFNGEMIDPFQLGLLVMFLATQEVLRKRWTARLTIPAVLGALLVLMLGGITYTDLARYVVLVAAAFAEANSGGDVLHLALIAVFKIQPAFLVMNMLSARWTNQENVVLVLGAAFFQLASVDLQIGVHGILNAAAIAWMIVRAITFPTTSTVAMPILALLTPGMRALYLDTYRIILLVIGICSLLQERRKTMAKKKGAVLLGLALTSTGWFSPTTIAAGLMVCNPNKKRGWPATEFLSAVGLMFAIVGGLAELDIESMSIPFMLAGLMAVSYVVSGKATDMWLDRAADISWEMEAAITGSSRRLDVKLDDDGDFHLIDDPGVPWKVWLLRMSCIGLAALTPWAIVPAAFGYWLTLKTTKRGGVFWDTPSPKPCLKGDTTTGVYRIMARGILGTYQAGVGVMYENVFHTLWHTTRGAAIMSGEGKLTPYWGSVKEDRISYGGPWRFDRKWNGTDDVQVIVVEPGKPAVNIQTKPGVFRTPFGEVGAVSLDYPRGTSGSPILDSNGDIIGLYGNGVELGDGSYVSAIVQGDRQEEPVPDAYTPSMLKKRQMTVLDLHPGSGKTRKILPQIIKDAIQQRLRTAVLAPTRVVAAEMAEALRGLPVRYQTSAVQREHQGNEIVDVMCHATLTHRLMSPNRVPNYNLFVMDEAHFTDPASIAARGYIATKVELGEAAAIFMTATPPGTTDPFPDSNAPIHDLQDEIPDRAWSSGYEWITDYAGKTVWFVASVKMGNEIAMCLQRAGKKVIQLNRKSYDTEYPKCKNGDWDFVITTDISEMGANFGASRVIDCRKSVKPTILEEGEGRVILGNPSPITSASAAQRRGRVGRNPNQVGDEYHYGGATSEDDSNLAHWTEAKIMLDNIHMPNGLVAQLYGPEREKAFTMDGEYRLRGEEKKNFLELLRTADLPVWLAYKVASNGIQYTDRKWCFDGPRTNAILEDNTEVEIVTRMGERKILKPRWLDARVYADHQALKWFKDFAAGKRSAVSFIEVLGRMPEHFMGKTREALDTMYLVATAEKGGKAHRMALEELPDALETITLIVAITVMTGGFFLLMMQRKGIGKMGLGALVLTLATFFLWAAEVPGTKIAGTLLVALLLMVVLIPEPEKQRSQTDNQLAVFLICVLTVVGVVAANEYGMLEKTKADLKSMFGGRTQAPGLTGLPSMALDLRPATAWALYGGSTVVLTPLLKHLITSEYVTTSLASISSQAGSLFVLPRGVPFTDLDLTVGLVFLGCWGQITLTTFLTAMVLVTLHYGYMLPGWQAEALRAAQRRTAAGIMKNAVVDGMVATDVPELERTTPLMQKKVGQVLLIGVSVAAFLVNPNVTTVREAGVLVTAATLTLWDNGASAVWNSTTATGLCHVMRGSYLAGGSIAWTLIKNADKPSLKRGRPGGRTLGEQWKEKLNAMSRDEFFKYRREAIIEVDRTEARRARRENNIVGGHPVSRGSAKLRWLVEKGFVSPIGKVIDLGCGRGGWSYYAATLKKVQEVKGYTKGGAGHEEPMLMQSYGWNLVSLKSGVDVFYKPSEPSDTLFCDIGESSPSPEVEEQRTLRVLEMTSDWLHRGPREFCIKVLCPYMPKVIEKMEVLQRRFGGGLVRLPLSRNSNHEMYWVSGAAGNVVHAVNMTSQVLLGRMDRTVWRGPKYEEDVNLGSGTRAVGKGEVHSDQKKIRKRIQKLREEFATTWHKDPEHPYRTWTYHGSYEVKATGSASSLVNGVVKLMSKPWDAIANVTTMAMTDTTPFGQQRVFKEKVDTKAPEPPAGVKEVLNETTNWLWAHLSREKRPRLCTKEEFIKKVNSNAALGAVFAEQNQWSTAREAVGDPLFWEMVNEERENHLRGECHTCVYNMMGKREKKPGEFGKAKGSRAIWFMWLGARYLEFEALGFLNEDHWLSRENSGGGVEGSGVQKLGYILRDIAGKQGGKMYADDTAGWDTRITRTDLENEAKVLELLDGEHRMLARAIIELTYRHKVVKVMRPAAGGKTVMDVISREDQRGSGQVVTYALNTFTNIAVQLVRLMEAEGVIGPQHLEQLPRKNKIAVRTWLFENGEERVTRMAISGDDCVVKPLDDRFATALHFLNAMSKVRKDIQEWKPSHGWHDWQQVPFCSNHFQEIVMKDGRSIVVPCRGQDELIGRARISPGAGWNVKDTACLAKAYAQMWLLLYFHRRDLRLMANAICSAVPVDWVPTGRTSWSIHSKGEWMTTEDMLQVWNRVWIEENEWMMDKTPITSWTDVPYVGKREDIWCGSLIGTRSRATWAENIYAAINQVRAIIGKENYVDYMTSLRRYEDVLIQEDRVI
