# Supplementary material for: Experimental Infection of Newly Hatched Domestic Ducklings via Japanese Encephalitis Virus-Infected Mosquitoes
Source: Pathogens. 2020 May 12;9(5):371. doi: 10.3390/pathogens9050371 (PMC7281460; doi:10.3390/pathogens9050371)
Supplement: Supplementary file 1 [file pathogens-09-00371-s001.zip › supplement materials/Table S1 Individual data of the ducklings exposed to JEV.docx]

**Table S1** Individual data of the ducklings exposed to JEV-infected *Cx. pipiens* mosquitoes

| Number | Level of RNAemia (copies of E gene/0.1 ml) | | | | Clinical response | | | | Note |
| --- | --- | --- | --- | --- | --- | --- | --- | --- | --- |
|  | 2 dpi | 3 dpi | 4 dpi | 5 dpi | Vague | Anorexia | Opisthotonos | Survive |  |
| 1 | 35843 | 436698 | 74032 | 239 | No | No | No | 🗸 |  |
| 2 | 25577 | NA | NA | NA | Yes | Yes | Yes | 🗴 | Pathological examination, viral detection and isolation |
| 3 | ND* | ND | 22457 | 767 | No | No | No | 🗸 |  |
| 4 | 21759 | 27794 | ND | ND | No | No | No | 🗸 |  |
| 5 | 38609 | 383115 | NA | NA | No | Yes | Yes | 🗴 | Pathological examination, viral detection and isolation |
| 6 | 27058 | ND | ND | 546 | No | No | No | 🗸 |  |
| 7 | ND | 19620 | 76248 | 51 | No | No | No | 🗸 |  |
| 8 | 33901 | 60766 | 78514 | 723 | No | No | No | 🗸 |  |
| 9 | ND | 15602 | 144454 | ND | No | No | No | 🗸 |  |
| 10 | 25533 | 27766 | ND | ND | No | No | No | 🗸 |  |
| 11 | 19788 | NA | NA | NA | Yes | Yes | Yes | 🗴 |  |
| 12 | NA | NA | NA | NA | No | No | Yes | 🗴 |  |
| 13 | 39977 | ND | 102392 | 1499 | No | No | No | 🗸 |  |
| 14 | 34779 | ND | ND | ND | No | No | No | 🗸 |  |
| 15 | 29012 | 18716 | NA | NA | Yes | No | Yes | 🗴 | Pathological examination, viral detection and isolation |
| 16 | ND | 30534 | ND | ND | No | No | No | 🗸 |  |
| 17 | ND | ND | 156108 | 2867 | No | No | No | 🗸 |  |
| 18 | 19393 | NA | NA | NA | Yes | Yes | Yes | 🗴 |  |
| 19 | 59482 | 42682 | ND | ND | No | No | No | 🗸 |  |
| 20 | 34964 | 25359 | ND | ND | No | No | No | 🗸 |  |

*, ND = not done. NA = Not available because ducklings died.
