# Supplementary figures and images for: Experimental Infection of Newly Hatched Domestic Ducklings via Japanese Encephalitis Virus-Infected Mosquitoes
Source: Pathogens. 2020 May 12;9(5):371. doi: 10.3390/pathogens9050371 (PMC7281460; doi:10.3390/pathogens9050371)

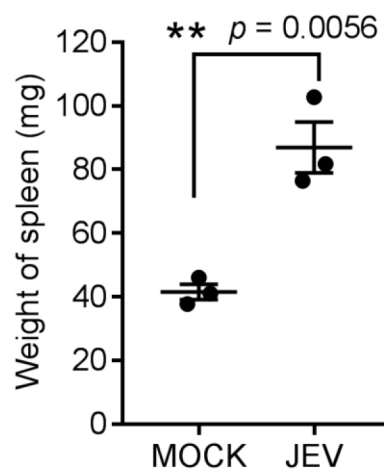

(a)

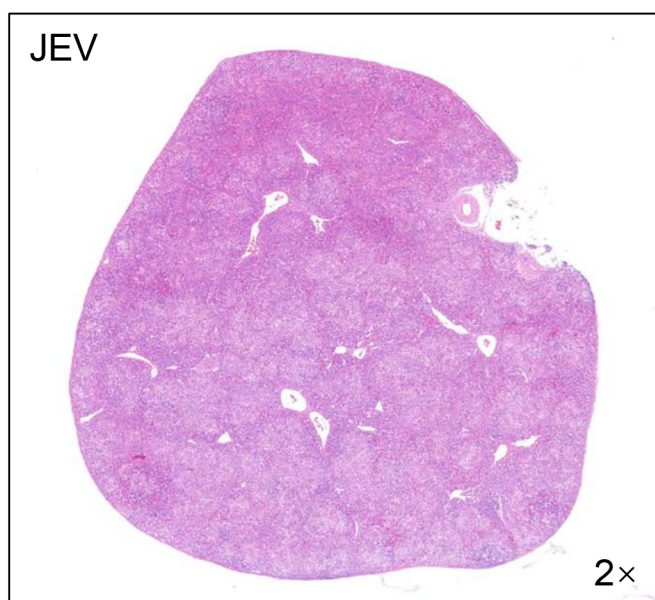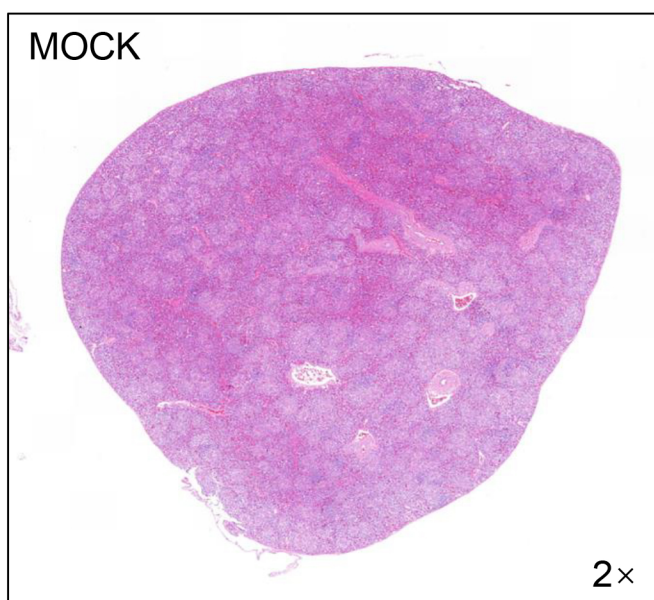

(b)

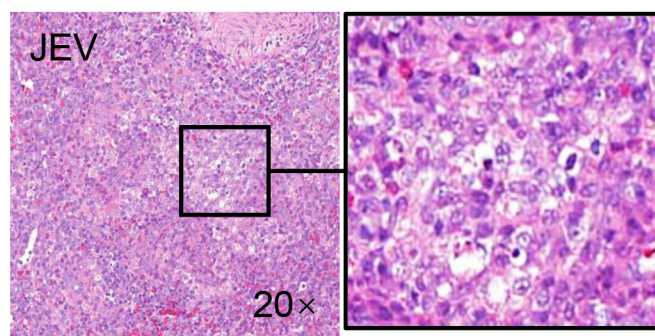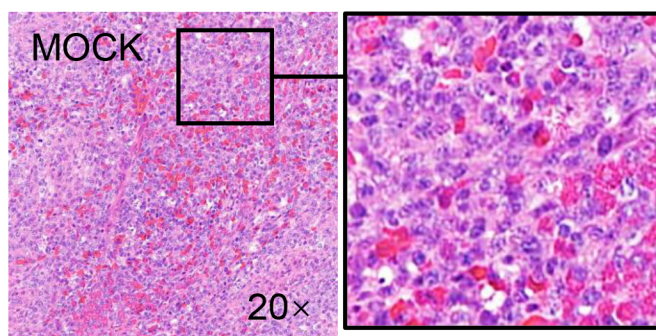

(c)

Supplement: Supplementary file 1 [file pathogens-09-00371-s001.zip › supplement materials/Figure S1.pdf]
